# KPC-33 and ompK37 mutations: Unraveling the mechanism of ceftazidime/avibactam resistance in ST11 carbapenem-resistant *Klebsiella pneumoniae*

**DOI:** 10.1371/journal.pone.0342729

**Published:** 2026-03-02

**Authors:** Shulong Zhao, Lin Ye, Shuang Song, Jingfang Sun, Jinfeng Xu, Fei Jiang, Haiquan Kang

**Affiliations:** 1 Department of Laboratory Medicine, Affiliated Hospital of Xuzhou Medical University, Xuzhou, Jiangsu, China; 2 Medical Technology School, Xuzhou Medical University, Xuzhou, Jiangsu, China; 3 Department of Clinical Laboratory, Zhenjiang Center for Disease Control and Prevention, Zhenjiang, Jiangsu, China; Ahvaz Jondishapour University of Medical Sciences Faculty of Medicine, IRAN, ISLAMIC REPUBLIC OF

## Abstract

**Background:**

Global surveillance indicates rising ceftazidime-avibactam resistance among carbapenem-resistant *Klebsiella pneumoniae* (CRKP), with sequence type 11 predominating in China. The contribution of *bla*_KPC_ variants and porin alterations to high-level resistance remains significant.

**Methods:**

We employed whole-genome sequencing and functional analyses to characterise a CZA-resistant ST11 CRKP isolate recovered from a patient without prior exposure to ceftazidime-avibactam or carbapenems.

**Results:**

The isolate harboured *bla*_KPC-33_ on a non-conjugative plasmid and multiple non-synonymous mutations in the porin gene ompK37, concomitant with high-level resistance to ceftazidime-avibactam and carbapenems while retaining susceptibility to tigecycline, polymyxin B and amikacin.

**Conclusions:**

*bla*_KPC-33_ coupled with OmpK37 alterations underpins dual resistance to ceftazidime-avibactam and carbapenems in ST11 CRKP, underscoring the need for genomic surveillance and rapid detection of this resistance mechanism.

## 1 Introduction

Carbapenem-resistant *Klebsiella pneumoniae* (CRKP) producing KPC enzymes have disseminated globally, leaving ceftazidime-avibactam (CZA) as one of the last viable options. Nevertheless, prospective surveillance shows that CZA resistance among CRKP has been on the rise in recent years. This emerging resistance poses a significant threat to clinical treatment outcomes, as it limits the already scarce therapeutic choices for infections caused by CRKP. The mechanisms underlying CZA resistance in CRKP are complex and multifaceted, involving various genetic alterations. Understanding these mechanisms is crucial for developing effective strategies to combat the spread of CZA-resistant CRKP strains. [1,2]. Molecular epidemiology indicates that *bla*_KPC-2_/*bla*_KPC-3_ variants coupled with porin loss (OmpK35/OmpK37) are the dominant resistance drivers [3]; however, the contribution of the recently described *bla*_KPC-2_/*bla*_KPC-33_ allele remains under-characterised, especially in China where sequence type 11 (ST11) constitutes the majority of CRKP isolates [4].

*Bla*_KPC-33_ differs from *bla*_KPC-2_ by a single Asp179Tyr substitution that restores carbapenem hydrolysis while conferring CZA resistance in vitro [5]. Clinical isolates carrying *bla*_KPC-33_ have been reported in adult and paediatric patients receiving CZA, suggesting that prior use of CZA may select for this variant [6]. Additionally, cumulative data imply that triple non-synonymous substitutions in OmpK37 (Ile70Met, Ile128Met, Asn230Gly) further reduce CZA uptake and potentiate the resistant phenotype [7]. Despite these insights, no study has integrated whole-genome analysis with detailed MIC data to dissect the synergistic effect of *bla*_KPC-33_ and OmpK37 mutations in ST11 CRKP.

Here, we employed hybrid long-read sequencing to characterise a CZA-resistant ST11 CRKP recovered from a patient without previous CZA or carbapenem exposure. Our findings delineate the genetic context of *bla*_KPC-33_ and quantify the impact of OmpK37 alterations on antimicrobial susceptibility, providing an evidence base for updated diagnostic and surveillance strategies in China.

## 2 Materials and methods

### 2.1 Bacterial isolation and identification

A carbapenemase-producing *Klebsiella pneumoniae* strain, designated 2842, was isolated from a sputum sample collected in April 2023 from a 62-year-old male patient with severe traumatic brain injury at the Affiliated Hospital of Xuzhou Medical University, Xuzhou, China. The patient had received ceftazidime (2 g q8 h, 9 days) and cefoperazone–tazobactam (2 g q12 h, 5 days) during the preceding two weeks, but no ceftazidime–avibactam or carbapenems. Species identification was performed by Matrix-Assisted Laser Desorption/Ionization Time-of-Flight Mass Spectrometry (MALDI-TOF MS, Bruker Biotyper)

### 2.2 Antimicrobial susceptibility testing

MICs were determined by broth microdilution following CLSI M100-34th (2024) and EUCAST v14.0 (2024) breakpoints. Tigecycline MIC was determined by broth microdilution and interpreted using FDA breakpoints.Plates were incubated at 35 °C in ambient air for 16–20 h. Quality control was performed daily with *Escherichia coli* ATCC 25922 and *Klebsiella pneumoniae* ATCC 700603; all QC values fell within published ranges.

As only one clinical isolate was analysed, no inferential statistics were applied; MIC values are reported descriptively without median/IQR calculations or inter-group comparisons.

### 2.3 Whole-genome sequencing and analysis

Genomic DNA was extracted using a commercial kit (Novogene). Illumina MiSeq (2 × 150 bp) and PacBio Sequel II (HiFi) libraries were constructed and sequenced by Novogene (Beijing, China). Hybrid de novo assembly was performed with Unicycler v0.4.8 (default parameters); mean coverages were 220× (Illumina) and 65× (PacBio) for the chromosome, and 180–350× for the plasmids. High-quality reads (mean Phred score Q > 35) were retained to keep base error rate <0.05% [8]. MLST was assigned using the Pasteur scheme (https://bigsdb.pasteur.fr). Plasmid replicons and resistance genes were identified with PlasmidFinder and ResFinder 4.1 (Center for Genomic Epidemiology), respectively. Genome annotation was carried out using the NCBI PGAP pipeline. The complete chromosome and plasmid sequences were deposited under NCBI BioProject PRJNA1161147 (accessions CP170134–CP170136). Raw Illumina and PacBio reads are available in the Sequence Read Archive (SRR35805130,SRR35805131) under the same BioProject;

### 2.4 Conjugation experiment

A conjugation experiment was conducted to ascertain whether the *bla*_KPC-33_ gene was located on a conjugative plasmid. *K. pneumoniae* 2842 served as the donor, and *E. coli* J53 as the recipient. Transconjugants were selected on LB agar plates supplemented with sodium azide (100 µg/ml) and meropenem (0.3 µg/ml). The presence of the resistance gene *bla*_KPC-33_ in transconjugants was confirmed by PCR, following the methods described in the study.

As a positive control, *K. pneumoniae* ATCC BAA-1705 carrying *bla*_KPC-2_ on an IncFII plasmid was mated with E. coli J53, yielding transconjugants at a frequency of 10 ⁻ ⁵. Heat-killed donor cells (100 °C, 15 min) served as the negative control and produced no growth on selection plates. Strain 2842 gave no transconjugants (<10 ⁻ ⁹), consistent with the absence of tra genes on p2842-1.

### 2.5 Ethical approval

This study was approved by the Ethics Committee of the Affiliated Hospital of Xuzhou Medical University (XYFY2022-KL008–01) and informed consents were obtained from all patients, available upon written request.

## 3 Results and discussion

### 3.1 Resistance profile of *K. pneumoniae* 2842

Two weeks before the isolate was obtained, the patient had received ceftazidime (2 g q8h, 9 days) and cefoperazone/tazobactam (2 g q12h, 5 days), providing a plausible selective window for *bla*_KPC-33_ emergence.

Strain 2842 exhibited high-level resistance to ceftazidime-avibactam (MIC ≥ 32 mg/L), imipenem and meropenem (MIC ≥ 16 mg/L each), while remaining susceptible to tigecycline and polymyxin B (**Table 1**). A complete MIC panel for 21 antimicrobial agents is provided in S1 File.

**Table 1:** Minimum inhibitory concentrations (MICs) and interpretive categories of antimicrobial agents against *Klebsiella pneumoniae* strain 2842.

NO.	Antimicrobial agent	MIC (µg/mL)	Interpretation*
01	Ceftazidime-avibactam	>32	R
02	Imipenem	>16	R
03	Meropenem	>16	R
04	Ciprofloxacin	>4	R
05	Tigecycline	<0.5	S
06	Polymyxin B	<0.5	S
07	Amikacin	<2	S
08	Cefotaxime	>64	R

*R = resistant; I = intermediate; S = susceptible. Breakpoints from CLSI M100-34th (2024) [9] and EUCAST v14.0 (2024) [10].Tigecycline MIC was determined by broth microdilution and interpreted using FDA breakpoints.

Although *bla*_KPC-33_ typically restores carbapenem susceptibility while conferring CZA resistance [5], 2842 retained full carbapenem resistance, creating a “camouflage” phenotype that can be missed by routine meropenem-based screening. Epidemiological data show that the patient had never received CZA, indicating that ceftazidime or cefoperazone/tazobactam can select *bla*_KPC-33_ in vivo—a scenario rarely documented previously.To place the present isolate in context, Table 2.

**Table 2:** Comparison of recently reported ST11 CRKP isolates exhibiting ceftazidime-avibactam resistance.

Strain (country, year)	ST	*bla*_KPC_ allele	Prior CZA†	CZA MIC^‡^ (mg/L)	Reference
KPHRJ (China, 2022)	11	KPC-33	YES	32	[7]
ppKP697_3(China, 2021)	11	KPC-71	YES	32	[11]
ST11-KL19(China, 2025)	11	KPC-207/KPC-208	YES	32	[12]
LX02 (China, 2025)	11	KPC-190	YES	64	[13]
CRE146(China, 2023)	11	KPC-228	YES	32	[14]
2842 (China, 2023) **this study**	11	KPC-33	NO	32	Present study

† Prior CZA exposure was extracted from electronic pharmacy records or published case reports; “YES” indicates ≥1 dose of ceftazidime-avibactam administered within 90 days before isolate collection.

‡ MICs determined by broth microdilution.

### 3.2 Whole-genome characteristics

Illumina clean reads (11,578,488; 150PE) and PacBio RS reads (120,568; mean 14.9 kb) were assembled into a single chromosome (5,451,441 bp; G + C 57.25%) and two plasmids: p2842-1 (91,212 bp; G + C 53.77%) and p2842-2 (261,134 bp; G + C 47.68%). Annotation (NCBI PGAP) revealed 5,561 CDS, 78 tRNAs, 8 rRNAs, 1 tmRNA and 129 pseudogenes. MLST assigned ST11, a high-risk clone endemic in China.A complete list of resistance determinants and key mutations detected in strain 2842 is provided in **Table 3**.

**Table 3:** Resistance determinants detected in *Klebsiella pneumoniae* 2842.

	Gene or protein	Identity (%) or amino acid change(s)	Antibiotic resistance	Location	Best-hit GenBank accession no.
*Genes*	*aadA2b*	99.87%	streptomycin’, ‘spectinomycin’	Chromosome	D43625
	*bla*_SHV-11_	99.88%	beta-lactam	Chromosome	JX121125
	*fosA6*	98.81%	Fosfomycin	Chromosome	KU254579
	*sul1*	100.00%	sulfamethoxazole	Chromosome	AY522923
	*bla*_KPC-33_	100.00%	Ceftadine/Avibatam ‘amoxicillin’, ‘amoxicillin+clavulanic acid’, ‘ampicillin’, ‘ampicillin+clavulanic acid’, ‘cefepime’, ‘cefotaxime’, ‘cefoxitin’, ‘ceftazidime’, ‘ertapenem’, ‘imipenem’, ‘meropenem’, ‘piperacillin’, ‘piperacillin+tazobactam’, ‘aztreonam’, ‘ticarcillin’, ‘ticarcillin+clavulanic acid’	p2842-1	CP025144
	*catA2*	96.11%	‘chloramphenicol	p2842-1	X53796
	*aac(3)-IId*	99.88%	‘gentamicin’, ‘tobramycin’, ‘dibekacin’, ‘netilmicin’, ‘apramycin’, ‘sisomicin	p2842-2	EU022314
	*mph(A)*	100.00%	erythromycin’, ‘azithromycin’, ‘spiramycin’, ‘telithromycin	p2842-2	D16251
	*bla*_TEM-1B_	100.00%	‘amoxicillin’, ‘ampicillin’, ‘piperacillin’, ‘ticarcillin’, ‘cephalothin’	p2842-2	AY458016
Protein	OmpK37	I70M, I128M N230G	Carbapenem	Chromosome	AJ011502.1
	acrR	P161R G164A F172S R173G L195V F197I K201M	fluoroquinolone	Chromosome	AJ318073.1

Gene symbols are italicized; protein symbols are in roman type. Identity determined by ResFinder 4.1 (≥90% nucleotide identity and ≥60% coverage).

### 3.3 Plasmid analysis

p2842-1 (IncFII/IncR) carries *bla*_KPC-33_ and *catA2* within an IS26-flanked 90.7-kb island (Tn3-IS26-ISKpn6-*bla*_KPC-33_-ISKpn27-tnpR-IS26-*catA2*-IS26) and shares 99.96% identity with pF5/pF5_1 (CP016403). Absence of *tra* genes concurs with negative conjugation.

**Fig 1:**
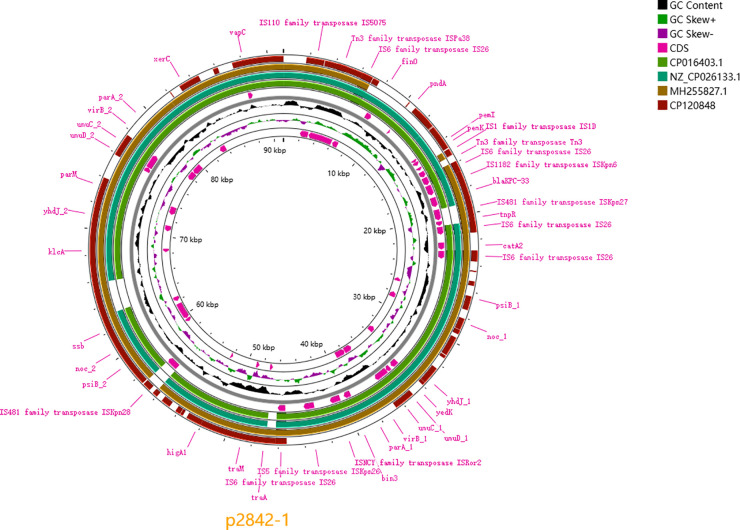
Circular comparison of p2842-1 (91 kb) carrying *bla*_KPC-33_ with pF5/pF5_1, pSH9-KPC and pKPHRJ. Map generated with Proksee.

**Fig 2:**
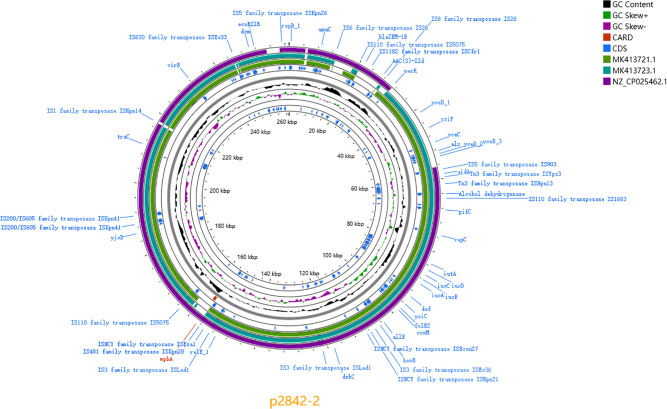
Circular comparison of p2842-2 (261 kb) carrying *aac(3)-IId*, *mph(A)* and *bla*_TEM-1B_ with p504051-HI3/p721005-HI3 and p44-1. Map generated with Proksee.

**Fig 3:**
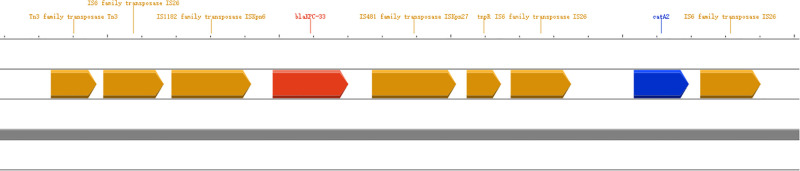
The red arrow indicates *bla*_KPC-33_; yellow arrows, IS26/ISKpn6 elements; blue arrows, replication/partition proteins; grey shading. The diagram illustrates the chromosomal integration hotspot of the IS26-flanked resistance module. Map was generated with Proksee.

p2842-2 (IncFIB/IncHI1B) harbours *bla*_TEM-1B_, *AAC(3)-IId* and *mphA* and is 99.87% identical to p504051-HI3/p721005-HI3. Both plasmids lack complete conjugative modules (Figs 1-2).The chromosomal context (±50 kb) surrounding the integrated *bla*_KPC-33_ module is illustrated in Fig 3.

### 3.4 Discussion

We document a ST11 *Klebsiella pneumoniae* co-harbouring *bla*_KPC-33_ and triple OmpK37 substitutions (I70M+I128M + N230G) in Jiangsu Province, China. The strain displayed “camouflage” resistance—ceftazidime-avibactam (CZA) MIC ≥ 32 mg/L while carbapenem MICs remained ≥16 mg/L, contradicting previous reports that Asp179Tyr restores imipenem susceptibility [6]. This phenotypic inconsistency highlights the necessity of combining porin loss with enzyme characterization when interpreting MIC data.

This combination yields CAZ-AVI MIC ≥ 32 mg/L in the present ST11 isolate, whereas previously reported KPC-33-harbouring strains remained susceptible at 2–8 mg/L [5], demonstrating a phenotypic escalation beyond the enzyme-only effect.Asp179Tyr expands the Ω-loop of KPC-2, enlarging the acyl-enzyme pocket and increasing catalytic efficiency against avibactam 15-fold relative to KPC-2 [15]. The triple OmpK37 substitutions (I70M/I128M/N230G) narrow the porin channel and are associated with reduced outer-membrane permeability to carbapenems, cooperating with KPC-33 to confer ceftazidime-avibactam resistance [16,17], providing a physical barrier that keeps carbapenem MICs elevated even in the presence of a less efficient carbapenemase. Thus, enzyme kinetics plus reduced influx synergistically generate the dual resistance phenotype.

Routine meropenem-based screening failed to flag the isolate as a carbapenemase producer, consistent with recent observations that *bla*_KPC-33_ carriers are often missed by automated systems owing to low-level carbapenem resistance phenotypes.We therefore recommend (i) supplementing current algorithms with CZA gradient strips and (ii) adding a *bla*_KPC-33_-specific real-time PCR to the national AMR sentinel protocol. In particular, excessive or prolonged use of third-generation cephalosporins (e.g., ceftazidime, cefoperazone) exerts strong selective pressure in vivo and has been associated with a higher risk of colonisation or infection by carbapenem-resistant Enterobacterales, including *bla*_KPC_ harbourers [18–21]; strict β-lactam stewardship—especially avoiding prolonged monotherapy—should therefore be prioritised in wards where ST11 CRKP is endemic.

Although therapeutic options against CRKP remain scarce, emerging gene-editing tools offer a potential breakthrough for halting the horizontal transfer of resistance determinants. CRISPR-Cas9 systems have been demonstrated to selectively excise *bla*_KPC_ and *bla*_NDM_ plasmids, curing carbapenem resistance and blocking conjugative transfer in Enterobacteriaceae [22]. Sublethal antibiotic stress increases IS26 transposition activity, and CRISPR-based or antisense RNA targeting of IS26 transposase can curb resistance island rearrangements in *Klebsiella pneumoniae* [23]. The IS26-flanked module carried by p2842-1 in the present study (Tn3-IS26-ISKpn6-*bla*_KPC-33_-ISKpn27-tnpR-IS26-catA2-IS26) constitutes an ideal target for such interventions. Future studies should verify in vitro (i) the efficacy of these strategies in selectively eliminating KPC-33–OmpK37 dual-resistant *Klebsiella pneumoniae* and (ii) their impact on the gut microbial ecosystem. Implementing these approaches could open new avenues for the precise control of ST11 CRKP in China.

### 3.5 Limitations

#### 3.5.1 Single-isolate generalisability.

This study characterised only one clinical isolate, which limits the statistical power to estimate the prevalence of *bla*_KPC-33_ plus triple OmpK37 substitutions in China.

*Impact*: Prevalence figures cannot be extrapolated to provincial or national populations.

*Mitigation*: We will continue to monitor this evolving resistance pathway through ongoing multi-centre surveillance and targeted sequencing of ST11 CRKP isolates.

#### 3.5.2 Lack of functional complementation.

Permeability and efflux contributions were inferred *in silico*; isogenic knock-ins or porin electrophoresis were not performed.

*Impact*: The relative contribution of each OmpK37 substitution to ceftazidime-avibactam MIC remains correlative.

*Mitigation*: We will conduct targeted experimental work, including allelic exchange and porin electrophoresis, to quantify the individual contribution of each OmpK37 substitution to ceftazidime-avibactam susceptibility.

### 3.6 Conclusions

This work documents high-level CZA and carbapenem resistance in ST11 *K. pneumoniae* driven by *bla*_KPC-33_ plus OmpK37 triple substitutions on a non-conjugative IncFII/IncR plasmid. The camouflage phenotype underscores the need to include CZA gradient tests or *bla*_KPC-33_ PCR in central-China AMR surveillance.

## Supporting information

S1 File Minimum inhibitory concentration (MIC) raw data for 21 antimicrobial agents. (XLSX)
